# Local Anæsthesia

**Published:** 1889-10

**Authors:** R. B. White

**Affiliations:** Ennis, Texas


### LOCAL ANÆSTHESIA.

BY R. B. WHITE, M. D., ENNIS, TEXAS.

Written for the Courier-Record of Medicine and
Read before the North Texas Medical Association

The discovery of cocaine marks the beginning of a new era in the history of local anæsthesia, and though a variety of means can be used for the purpose, none of them will bear comparison with this agent.

Most general practitioners remember how reluctantly in former times they undertook even trifling operations on the eye, ear, nose, etc., in which as at present by taking advantage of cocaine anæsthesia, a considerable amount of this kind of work which used to be sent to the city specialist, can be done by the ordinary attendant. The saving to the patient in time and money by this course is often considerable, while it enables the family physician to so familiarize himself with eye-work, that he can safely undertake operations, thereby saving eyes which, had the distant occulist to be alone relied upon, would be lost for want of time. Every practitioner of experience can recall instances of this kind. In glaucoma especially, the delay of twenty four, or even of twelve hours, will often make all the difference between saved vision and permanant blindness. Dr. Gregory of St. Louis used to tell us—students of the St. Louis medical college—that he would never vote for conferring a diploma on any man unable to make an iridectomy or operate for strangulated hernia.

Not a few cutting operations can be made painlessly and certainly with greater safety to the patient under cocaine than under ether or chloroform. Under cocaine I have done circumcision, radical operation for hydrocele, removal of needle from hand, operation for lacera, ted perineum, various operations on rectum and genito-urinary organs-for varicose veins, and incisions of all kinds.

For the eye, ear throat, and mucous surfaces generally, the direct application of a four or five per cent solution is all that is requisite when the knife, needle or trochar has to be used; an amount varying from ten to twenty m. of the same solution is injected hypodermically into the tissues at the site of the operation. By using the proper kind of needle (and every one who practices surgery ought to be provided with a special cocaine point for hypodermic syringe) even the preliminary injection of cocaine is almost painless. Late works on surgery, as Wyeth's, fully describe the technique employed.

A fair degree of local anæsthesia can be obtained, when from any cause the surgeon does not desire to use cocaine, by the use of ice and salt, the ether spray, rhigoline, and by causing the patient to breathe rapidly, say sixty to eighty respirations per minute. The last mentioned method we owe, I believe to Dr. Addinell Hewson of Philadelphia who fully described and demonstrated the procedure at the Centennial International Medical Congress held in that city in 1876. It is simply to make the patient breathe with great rapidity for about eighty seconds. It is generally necessary for the operator to start the patient by himself taking a few rapid respirations and urging the patient to imitate him. Very soon he will be breathing automatically, as it were, and though the resulting anæsthesia is transitory, yet it has sufficed in the writer's hands for opening abscesses, dilating sphincter ani by Van Buren's method, extracting teeth, etc. Some patients can not be induced to keep up rapid breathing long enough, but the exercise of a little tact and will-power on the part of the surgeon will generally succeed with even stupid and intractable patients.

Cocaine is, of course, the local anæsthetic *par excellence* and is generally safe, but it must not be overlooked that even its careful and moderate use sometimes has brought on dangerous and alarming symptoms. On one occasion I almost killed a young man by applying an amount in solution equivalent to about 2/3 of a grain to an ulcerated rectum, as a preparatory step in dilating the sphincter. In five minutes he was completely prostrated, with respiration and pulse rapid and irregular, face and hands cold and bathed in perspiration. The prompt use of atropia hypodermically with inhalation of amyle nitrite, soon however, brought him around. A like train of symptoms sometimes, though rarely, follows the hypodermic use of cocaine. I know no instance in which death has actually resulted from the moderate and legitimate use of any local anæsthetic.

An ointment of cocaine using lanoline as a base is the proper mode for painless catheterization. Lard ought never to be used as the basis of cocaine ointment, as it in some way destroys or neutralizes the drug.

Since writing the foregoing I have been called on to adjust a fracture of forearm. My friend and colleague Dr. R. A. McCall of this city assisting.

We expected to use chloroform, but before its administration, decided to try cocaine; 125m. of the five per cent solution was injected deeply; the needle point could be felt impinging on fragments. Esmarchs bandage was applied above elbow so as to confine the cocaine laden blood. The result was all that could be desired. The patient, a boy of seven years, declared that our manipulations were absolutely painless.

